# Volume-related weight gain as an independent indication for renal replacement therapy in the intensive care units

**DOI:** 10.15171/jrip.2017.07

**Published:** 2016-11-06

**Authors:** Tibor Fülöp, Lajos Zsom, Mihály B. Tapolyai, Miklos Z. Molnar, László Rosivall

**Affiliations:** ^1^Department of Medicine, Division of Nephrology, University of Mississippi Medical Center, Jackson, MS, USA; ^2^FMC Extracorporeal Life Support Center, Fresenius Medical Care Hungary, Medical and Health Science Centre, University of Debrecen, Debrecen, Hungary; ^3^Division of Transplantation, Institute of Surgery, University of Debrecen, Debrecen, Hungary; ^4^Fresenius Medical Care, Semmelweis University, Budapest, Hungary; ^5^Carolinas Campus, Edward Via Osteopathic College of Medicine, Spartanburg, SC, USA; ^6^Division of Nephrology, Department of Medicine, University of Tennessee Health Science Center, Memphis, TN, USA; ^7^Department of Transplantation and Surgery, Semmelweis University, Budapest, Hungary; ^8^Institute of Pathophysiology, Semmelweis University, Budapest, Hungary

**Keywords:** Acute kidney injury, Biomarkers, Critical illness, Oliguria, Renal failure, Volume overload

## Abstract

Attempts to identify specific therapies to reverse acute kidney injury (AKI) have been unsuccessful in the past; only modifying risk profile or addressing the underlying disease processes leading to AKI proved efficacious. The current thinking on recognizing AKI is compromised by a "kidney function percent-centered" viewpoint, a paradigm further reinforced by the emergence of serum creatinine-based automated glomerular filtration reporting over the last two decades. Such thinking is, however, grossly corrupted for AKI and poorly applicable in critically ill patients in general. Conventional indications for renal replacement therapy (RRT) may have limited applicability in critically ill patients and there has been a relative lack of progress on RRT modalities in these patients. AKI in critically ill patients is a highly complex syndrome and it may be counterproductive to produce complex clinical practice guidelines, which are labor and resource-intensive to maintain, difficult to memorize or may not be immediately available in all settings all over the world. Additionally, despite attempts to develop reliable and reproducible biomarkers to replace serum creatinine as a guide to therapy such biomarkers failed to materialize. Under such circumstances, there is an ongoing need to reassess the practical value of simple measures, such as volume-related weight gain (VRWG) and urine output, both for prognostic markers and clinical indicators for the need for RRT. This current paper reviews the practical utility of VRWG as an independent indication for RRT in face of reduced urine output and hemodynamic instability.

**Implication for health policy/practice/research/medical education:**

Conventional indications for renal replacement therapy (RRT) may have limited applicability in critically ill patients. There is an ongoing need to reassess the practical value of simple measures, such as volume-related weight gain (VRWG) and urine output, both for prognosis and indications of RRT. This current paper reviews the practical utility of VRWG as an independent indication for RRT in face of reduced urine output and hemodynamic instability.

## Introduction

Acute kidney injury (AKI) (1) is common in sepsis and has been linked to compromised survival in this setting (2). Defining a truly effective treatment for AKI for these patients – beyond the most evident, that is appropriate antibiotics, volume replacement and general supportive measures – has been a series of controversies and failures. Clearly, early goal-directed therapy in sepsis has been driving the policy of aggressive volume resuscitation in intensive care units (ICUs) (3). However, an early study of successful goal-directed therapy to optimize mixed venous O_2_ saturation by Rivers et al (4), could not be replicated by large, multi-center trials (5,6). In both non-confirmatory trials, the intervention groups received slightly larger fluid volume but the difference in the amount of fluid given was unlikely to be biologically meaningful. We propose here that adequate but not excessive fluid resuscitation is a critical factor driving outcome. Thus, our paper aims to review the practical utility of volume (fluid) overload as an independent indication for renal replacement therapy (RRT), especially when such is considered as it is related to reduce urine output and hemodynamic instability.

## Materials and Methods

For this review, we performed a literature search through PubMed/Medline and Google Scholar, with attention to the literature published during years of 2010-2015, as well as early part of 2016. The search was conducted using combination of the following key words and or their equivalents; acute kidney injury, biomarkers, critical illness, oliguria, renal failure and volume overload. Titles and abstracts of review articles, case-control studies, clinical trials, cohort studies and reports that held relevance to the intended topic were studied. Bibliography of selected publications was additionally reviewed for relevant publications. Clinically experience of the authors was considered, when summing up and writing this review.

## Non-classic indications for RRT

AKI remains common in critically ill patients and is independently associated with increased morbidity, mortality and health care costs (7-9), especially in the context of multi-organ failure (10,11). Considerable effort has been disbursed into identifying prognostic factors and treatment variables that may predict or modify survival in these patients (12-19). Classic indications for RRT formulated for chronic dialysis may no longer fit the clinical expectations and disease burden of patients with critical illness and severe AKI in the ICU setting (20). While acute and severe hyperkalemia, metabolic acidosis not amenable to medical therapy or other electrolytes abnormalities may prompt emergent renal replacement in the ICU setting, these indications may or may not be present and may not represent the largest pressing clinical concern in critically ill patients with renal failure. Other indications, such as oliguria or volume overload with hemodynamic instability and respiratory failure are more commonly encountered and are more difficult to address. One emerging clinical parameter of great importance for practicing nephrologists and intensive care physicians is volume-related weight gain (VRWG) that is, the net isotonic fluid volume accumulating during the critical illness. Such volume overload confounds the elevation of serum creatinine via dilution of the extracellular fluid space and masks the true degree of vasodilatation-related hemodynamic instability. With a seemingly normal or close to normal blood pressure (BP), major volume overload (>10%-20% VRWG above baseline weight) is a very alarming sign for covert hemodynamic instability and the potential for tissue hypoxia. To state it differently, one would expect volume overloaded subjects to be hypertensive; the lack of such is, in fact, a “hypotension-equivalent” and a valuable clinical sign on its own merit.

Volume overload has been viewed historically in terms of posing danger as pulmonary edema and respiratory failure. While this may be the most conspicuous finding for respiratory care physicians, one relatively newly recognized pattern is the appreciation of elevated intra-abdominal pressures or frank abdominal compartment syndrome in the non-surgical ICU patients (21,22). Such entity has been long recognized for surgical ICU patients, especially when aggressive volume resuscitation had taken place (23). The elevated intra-abdominal pressure is thought to compromise arterial perfusion and venous return in the abdomen, especially in subjects with low mean arterial pressures. Accordingly, in abdominal compartment syndrome, the final common denominator is the net tissue perfusion pressure estimated from the difference of mean arterial pressure and intra-abdominal pressure (added to venous return pressure). This concept may shed light to one of the great conundrums of clinical medicine, i.e. the relative good success of acute peritoneal dialysis (PD) among critically ill patients as it is commonly practiced in relatively resource-limited environments (24-27). Placing a temporary PD catheter draining ascites that is part of the excessive volume accumulated in critically ill volume-overloaded patients enables abdominal decompression and thus, may reduce elevated intra-abdominal pressures and abort development of abdominal compartment syndrome. Accordingly, at least in our opinion, the success of acute PD in critical illness may not entirely hinge on the clearance provided, but on the ability to change intraabdominal volume-pressure relationships, ultimately improving intra-abdominal tissue perfusion.

In a recent meta-analysis of blood purification trials in AKI, only hemoperfusion, plasma exchange, and hemofiltration with hemoperfusion but not hemodialysis was associated with lower mortality in patients with sepsis (28). However, these results were mainly influenced by studies using polymyxin-B hemoperfusion in Japan. Clearly, increasing intensity of RRT beyond conventionally recommended doses failed to improve patient survival (27,29) –or perhaps the cohort risk profile was such that no further escalation of treatment intensity could improve survival (30) – underlying the contributions by clearance-independent factors. Patients with baseline chronic kidney disease (CKD) represent yet another clinical dilemma: having a different baseline, the time-course of serum creatinine elevation is less well demarcated and clinical outcomes as they relate to the development of AKI are more difficult to estimate than in pure AKI (16,31,32).

## Kidney function and volume overload

To some degree, the current thinking is a prisoner of an era, viewing integrity of renal homeostasis as “kidney function percent,” a thinking paradigm further reinforced by the emergence of automated glomerular filtration rate (GFR) calculations (e.g., Modification of Diet in Renal Disease and CKD-EPI formula-based equations). When calculating fractional decline of renal function, only filtration is considered assuming that decline of all other functions of the human kidney would be parallel to the decline of GFR – an assumption obviously not always taking place in terms of other indices, for instance, volume overload and hemodynamic status. Such thinking, however, applies even less to the setting of AKI in critically ill patients. As reasonable as progressive decline of GFR can be described in gradual CKD progression between the 15-60 mL/min/1.73 m^2^ GFR range, clinical experience shows it to be inadequate when trying to define trigger points for clinical uremia in a catabolic state or need for RRT for volume-related indications. An observed serum creatinine may have little relevance in a wasted subject with low muscle mass, generalized edema and prolonged respiratory failure with difficulty in weaning (33). Volume overload, when massive, may completely mask the rise of serum creatinine and or markedly underestimates the degree of renal functional impairment.

Another gross difference between CKD and AKI is in the interpretation of residual kidney function (RRF). While RRF is of enormous importance in chronically dialyzed subjects to provide anti-uremic effect and volume control, it is more of a prognostic marker in AKI. Recently, Koyner et al (34) demonstrated that one-time standardized dose of furosemide in those with early AKI was highly predictive of adverse patient outcomes, with a urine output of <200 mL in the ﬁrst 2 hours after furosemide administration being associated with ~85% sensitivity and speciﬁcity to progression to stage-3 AKI and subsequent poor outcomes. Not unreasonably, such approach is now labeled as the “furosemide stress test” but, in fact, only recycles and validates a time-honored and customarily performed medical practice by many practitioners. Perhaps the key message from these studies is the futility of repeated furosemide administrations in non-responders. In these scenarios, further administration of furosemide may be a surrogate for delaying initiation of effective RRT. It is in such context that the practical value of timed urine collections may need to be reappraised. While classic 24-hour urine collections may result in further delays and excessive complexity, short-time measurement of serum and urine creatinine after a 4-6 hours period may provide important information virtually within the same working day and may in fact afford a real-time monitoring of GFR. Additionally, it may provide additional information in situations of low muscle mass or volume overload, which are common occurrences in critically ill patients (33).

## Volume overload and survival

Early adult experience on fluid overload was primarily derived from the surgical literature, noting increased morbidity and mortality in volume-overloaded patients with acute respiratory distress syndrome (35,36), sepsis (37), and surgical ICU patients (38,39), while lesser fluid gains were associated with better outcomes in abdominal compartment syndrome (40,41) or after colon resection (38). Interestingly, the pediatric literature reporting on the importance of volume-related increases in morbidity and mortality predated experience from adult cohorts, e.g. those with fluid overload at the initiation of continuous RRT (CRRT) in whom hypervolemia was associated with increased mortality (8,13,14). While this may sound surprising at first, pediatric cohorts are in fact less likely to be “contaminated” by multiple comorbidities; accordingly, despite their smaller size, pediatrics cohorts may demonstrate biomedical signals with less ambiguity. Among adults, our group (42) was one of the firsts to describe the progressive risk of death with increasing fluid from a single-center cohort of patients: we demonstrated increased odds ratio (OR) to 2.62 (95% confidence intervals [CI]: 1.07-6.44) for mortality with VRWG ≥10% on univariate analysis further rising to 3.98 (95% CI: 1.01-15.75; *P<*0.049) with VRWG ≥20%. Both VRWG ≥10% (OR 2.71, *P<*0.040) and oliguria (OR 3.04, *P<*0.032) maintained a statistically significant association with mortality in multivariate models that included that clinical diagnosis of sepsis and Apache II score. Similarly, Payen et al examined the “Sepsis Occurrence in Acutely ill Patients” (SOAP) multi-center database (43) to evaluate the effect of positive fluid balance with outcomes in patients admitted to the ICU. They reported that in patients with AKI non-survivors had a more positive fluid balance than the survivors. The “Program to Improve Care in Acute Renal Disease” (PICARD) group (44) analyzed data from their cohort of critically ill adult patients with AKI and found that patients with fluid overload had a significantly higher mortality, irrespective of the modality of RRT. Among patients surviving acute illness, Heung et al documented that fluid overload at the beginning of RRT is associated with less renal recovery on long-term (45). The last half decade from 2010 on witnessed a flurry of investigations from adult cohorts similarly demonstrating the importance of VRWG in AKI. In a multi-center trial of Finnish ICUs, fluid overload was associated with an increased risk for 90-day mortality (OR 2.6) after adjusting for disease severity, time of RRT initiation, initial RRT modality, and sepsis (46). Similarly, in an Italian multicenter trial, increase mean fluid accumulation (adjusted hazard ratio [HR] 1.67 per L/day, 95% CI 1.33 to 2.09; *P<*0.001) and urine volume (adjusted HR 0.47 per L/day, 95% CI 0.33 to 0.67; *P<*0.001) were independent risk factors for 28-day mortality after adjustment for age, gender, diabetes, hypertension, diuretic use, non-renal SOFA and sepsis (47). Interestingly, diuretic use was associated with better survival in this population (adjusted HR 0.25, 95% CI: 0.12-0.52; *P<*0.001). In a very recent (2015) Chinese multi-center trial from Beijing, fluid overload was an independent risk factor for incident AKI (OR 4.508, 95% CI: 2.900-7.008, *P<*0.001) and increased the severity of AKI (48). Non-survivors with AKI had higher cumulative fluid balance during the first 3 days (2.77 [0.86–5.01] L versus 0.93 [−0.80 to 2.93] L, *P<*0.001) than survivors did. Multivariate analysis revealed that the cumulative fluid balance during the first 3 days was an independent risk factor for 28 day mortality (48). And, finally, the most recent findings from the Dose Response Multicentre Investigation on Fluid Assessment (DoReMIFA) study, both the severity and rapidity for fluid accumulation development represented independent risk factors for ICU mortality (49). Further, in this latter study, fluid accumulation proved more harmful in the presence of AKI and preceded the renal dysfunction. All these observations, however, have one major potential major error – confounding by indication, i.e., sicker patients were more likely to be hypotensive and received larger amount of fluids in an attempt to control hypotensive tendency. A systematic review and meta-analysis further confirmed these associations between fluid overload with mortality in AKI but found only a non-significant trend for an association of fluid overload with renal recovery (50). Modifying intravenous fluid composition used for volume resuscitation so far proved a failure. Literature emerging over the last one decade attempted to support the use of colloid solutions over crystalloid solutions for volume resuscitation; however, concerns emerged about the association between hydroxyethyl starch and increased propensity for AKI in patients with sepsis (51). Similarly, albumin use failed to confer any survival benefits in controlled clinical trials (52,53) and may have contributed to increased intracranial pressure in select individuals with brain trauma. It appears that the degree of net fluid gained (that is, the VRWG) is a stronger predictor of outcomes than the type of fluid administered during the early the course after admission.

To address hypotension and volume overload in anuric subjects, CRRT is often preferred to intermittent hemodialysis to better control VRWG (54). Historically, the better tolerance of CRRT vs. conventional intermittent HD was attributed exclusively to the prolonged nature of the former enabling more fluid removal over an extended period (55,56). Nonetheless, at least clinical experience suggests that the true merit of RRT is not its ability to remove large amounts of fluid but in doing so while provoking less hemodynamic instability. It appears that several mechanisms are at play by which CRRT may decrease a hypotensive tendency (in particular, during veno-venous hemofiltration) to afford net fluid removal (57). Early modalities of continuous therapies without built-in heater circuit likely provoked cooling with subsequent shivering and vasoconstriction, contributing to the improved BP on CRRT. Certainly, in the authors’ experience, it appears that the ability of CRRT to decrease hemodynamic instability is the primary process by which may improve outcomes. With successful source control, such as removing the source of infectious-inflammatory injury (e.g., abscess drained), we observed many times gradual improvement of BP after 4-6 hours, enabling the clinicians to start net fluid removal. Nonetheless, individual responses are tremendous and calls for astute clinical re-evaluations in these patients. With regard to expected tolerated fluid removal, to us, the most obvious rule appears to be the lack of such hard rule. Indeed, currently there is no apparent replacement for an astute clinician’s daily assessment and judgement to estimate expected tolerance of fluid removal.

## Biomarkers of renal injury

Despite more than a decade of unfulfilled promises and spent efforts, we are yet to see a true disruptive technology to replace conventional markers or a seasoned clinicians’ experience. Taking a prime example, newer biomarkers of AKI are yet to supersede a very imperfect but much simpler biochemical marker, the serum creatinine in clinical utility (58). The added costs of newer biomarkers for AKI are an additional concern, especially in resource-limited settings. The heterogeneity of clinical etiology for AKI is yet another challenge for biomarker-based diagnostic procedures. In many instances, the underlying etiology of AKI is unclear; in contrast, many biomarker-defined prognostic schemes have been derived from a specific disease cohort of patients. Only few studies used dedicated study adjudicators in attempt to develop rigorous, consensus-based clinical definitions to correlate the markers. Complexity is another problem. In a time-pressured busy environment any recommended prediction model should be either exceedingly simple or very easily available via the healthcare system computer and bioinformatics network. There is also a major internal limitation for renal biomarkers. While most of the biomarker studies are focused on kidney-derived enzymes or products, AKI is in fact a systemic illness in many instances, being only one feature of multiple organ failure syndrome. Accordingly, there is a major conceptual difference between the so called “renal angina” (59,60) and, for instance, the classic paradigm of myocardial infarction (MI). In MI, the prime cause of abnormality is the necrosis of myocardial tissues; in renal diseases, most commonly, AKI is a functional consequence of a systemic process. In an elegant series of limited autopsies performed immediately after death, Takasu et al. demonstrated only a limited degree of renal injury in the examined tissues, markedly disconnected from the profound functional impairment in septic patients before death (61).

## Urine output

In most clinical contexts, decreases in the urine output (oliguria) implies an acute hemodynamic process or an acute component of renal failure. Oliguria is an excellent independent risk factor predicting adverse renal outcomes (62) and has been part of the various staging schemes and definition of AKI, such us the Risk-Injury-Failure-Loss-End-stage (“RIFLE”) classification and Acute Kidney Injury Network staging. One key concern about using oliguria as an independent clinical parameter is the lack of adjustment for volume status. Oliguria will have a very different meaning in the setting of volume depletion, euvolemia or an already existing frank volume overload. One would never hesitate to respond to oliguria in a volume-depleted patient by giving fluids. Often, fluid is given in an attempt to simply reverse oliguria, an independent objective taken out of the context of a complex disease process. However, this may not always be the optimal answer, even in subjects seemingly responding initially to such a maneuver. Oliguria itself may be a sign of an overall clinical illness, such as an emerging sepsis. Excessive volume resuscitation in this instance may be counterproductive resulting in tissue hypoxia with massive volume overload and pulmonary edema with respiratory failure. Multiple technologies are emerging to assist clinicians in this setting including assessing inferior vena cava size, bioimpedance (63-65), assessing lung water content by ultrasound (66,67) and biochemical markers for volume overload, such as elevated B-type natriuretic peptide (64,68). New onset albuminuria is an another, often-ignored marker of kidney injury, implying compromised proximal tubular reabsorption of ﬁltered albumin by injured tubules (69).

## Putting it together – considering VRWG and urine output together

When all is said and done, the presence of low urine output cannot have the same meaning in an approximately euvolemic subject as opposed to one already markedly volume overloaded (>10 or 20% VRWG). In doing so, the scenario would be somewhat analogous for patients on mechanical ventilator support; under those circumstance, one would never interpret an arterial blood gas panel and oxygenation without knowing the partial pressure or percent of O_2_ on the inhaled gas. Another paradigm would be to report on BP or heart rate without reporting on presence or dose of vasoactive pressor agents. Both practice would be unacceptable and unlikely to survive the rigor of daily rounds with the attending physicians. Yet, in the current word of practice we do report on urine output without quantifying on the degree of salt-water overload, save the historical “one or two plus” terms (an anachronism itself, for a bed-bound patent!). It is ironic, however, how difficult it may prove to obtain reliable daily weight in ICU setting. Another concern is lack of adjustment for body size or surface. The presence of a very large fat tissue compartment will add to the body weight but may blur interpretation of correct urine output further. Unlike GFR, urine output is not customarily adjusted for body surface. Given the excess weight of some of our patients in North America (> 120 or even 150 kg) the oliguric mark of less than 0.5 (or even 0.3) mL/kg of body weight/hour can be crossed with surprisingly good urine production. Consider, for example, 150 kg person – would any of us truly consider a urine output of 75 mL/h as low or a sign of AKI? There is an unexplored potential for multi-phase BIA to measure fat-free tissue mass, and to adjust predicted and expected urine output for the fat-free space.

## Concluding remarks

Conceptually, before being lost in the plethora of various biomarkers or a myriad of derived hemodynamic parameters, clinical nephrologist should consider using and correctly interpreting basic parameters of critically ill patients with renal failure in ICU. Similar to urine output, VRWG should be considered as “renal vital sign,” incorporated into the rounding report and daily consideration of care decisions. Additional consideration should be given to developing exact technology for measuring VRWG and adjusting the presence of urine output for lean mass and VRWG in future studies. There is beauty in simplicity, after all.

## Authors’ contribution

TF developed the paper’s concept, performed the literature search, and wrote the first draft and also coordinated manuscript revisions and correspondence. LZs and MZM contributed to literature review and provided critical review of the manuscript. MBT and LR provided additional critical review and input during manuscript development. All authors participated in preparing the final draft of the manuscript, revisited the manuscript and critically evaluated the intellectual contents. All authors have read and approved the content of the manuscript and confirmed the accuracy or integrity of any part of the work.

## Conflicts of interest

The authors have no potential conflict of interest to report. The views and opinions expressed in this paper are solely of the Authors’ own and do not necessarily reflect the official views and opinions of the affiliated institutions.

## Ethical considerations

Ethical issues (including plagiarism, data fabrication, double publication) have been completely observed by the authors.

## Funding/Support

None.
